# Parasitic Infections as Reversible Hyper-IgE Phenocopies in Children with Markedly Elevated IgE: A Retrospective Cohort Study

**DOI:** 10.3390/children13050653

**Published:** 2026-05-07

**Authors:** Kazım Okan Dolu, İdan Fırat Unay, Yasemin Tepe, Hande Üçler Çınar, Çağla Karavaizoğlu, Himmet Haluk Akar

**Affiliations:** 1 Department of Pediatric Allergy and Immunology, Kanuni Sultan Süleyman Training and Research Hospital, Istanbul 34303, Türkiye; 2 Department of Pediatric Allergy and Immunology, Health Sciences University, Başakşehir Cam and Sakura City Hospital, Istanbul 34480, Türkiye

**Keywords:** children, eosinophilia, hyperimmunoglobulinemia E, parasitic infection, scabies

## Abstract

**Highlights:**

**What are the main findings?**

- Parasitic infections were identified in 24.4% of children with IgE ≥ 2000 IU/mL; median IgE reduction was 63.8% vs. 27.0% in parasitic versus non-parasitic groups (*p* < 0.001), with 8.26-fold higher odds of IgE normalization below 2000 IU/mL.
- All three inflammatory National Institutes of Health Hyper-IgE Syndrome (NIH-HIES) score components (IgE, eosinophilia, eczema) regressed significantly more often following resolution of parasitic etiology, constituting a reversible phenocopy of primary immune dysregulation.

**What are the implications of the main findings?**

- In populations with substantial parasitic prevalence, parasitological evaluation may be a useful consideration in children with markedly elevated IgE and indeterminate clinical scores, potentially reducing unnecessary genetic workup in selected cases.
- In socioeconomically diverse populations with ongoing rural-to-urban migration, parasitic infections should be considered in the differential diagnosis of markedly elevated IgE.

**Abstract:**

**Background/Objectives:** We aimed to evaluate the etiologic distribution of markedly elevated IgE (≥2000 IU/mL) and the association of parasitic infections with longitudinal IgE dynamics and National Institutes of Health Hyper-IgE Syndrome (NIH-HIES) score trajectories in pediatric patients. **Methods:** We retrospectively enrolled 127 pediatric patients with IgE ≥2000 IU/mL and ≥1 year of longitudinal follow-up at a tertiary allergy clinic (2019–2024). The primary outcome was the IgE reduction percentage difference between parasitic and non-parasitic groups. **Results:** Atopic sensitization was identified in 81.0% of tested patients; genetic evaluation was performed in 40 patients (31.5%), with confirmed IEI in one patient (0.8% of the cohort; 2.5% of those evaluated). Parasitic infections were present in 24.4% of patients (*n* = 31; intestinal parasites 15.7%, scabies 8.7%, hydatid cyst 1.6%; two patients had concurrent intestinal parasitosis and scabies). Median IgE reduction was 63.8% vs. 27.0% in parasitic versus non-parasitic groups (*p* < 0.001), persisting after multivariable adjustment (*p* < 0.001). Parasitic infection independently predicted IgE normalization below 2000 IU/mL (OR = 8.26; 95% CI: 2.76–24.68; *p* < 0.001). All three NIH-HIES inflammatory components (IgE, eosinophilia, eczema) regressed more often in the parasitic group (all *p* ≤ 0.01); no patient reached the ≥40-point HIES threshold. **Conclusions:** Parasitic infections produce a clinical phenotype overlapping with hyper-IgE syndrome, constituting a reversible phenocopy of primary immune dysregulation. In populations with substantial parasitic prevalence, parasitological evaluation may be a useful consideration in children with markedly elevated IgE and indeterminate clinical scores; however, this approach should complement rather than replace comprehensive clinical assessment.

## 1. Introduction

Markedly elevated serum total immunoglobulin E (IgE) is a frequently encountered but etiologically heterogeneous finding in pediatric allergy and immunology practice. Although atopic disorders account for the majority of cases, parasitic infections, chronic inflammatory conditions, hematologic malignancies, and inborn errors of immunity (IEI) may also result in substantial IgE elevation [1,2,3,4]. Accurate identification of the underlying etiology is essential for early diagnosis and for avoiding unnecessary diagnostic workup.

Because parasitic infections share immunological features with IEI—particularly elevated IgE, eosinophilia, and eczema—they may mimic IEI in clinical scoring systems, potentially leading to unnecessary genetic evaluation. This study aimed to describe the etiologic distribution in pediatric patients with serum total IgE ≥ 2000 IU/mL and to evaluate the longitudinal course of IgE, eosinophil, and NIH-HIES score components in parasitic versus non-parasitic groups.

## 2. Materials and Methods

This retrospective cohort study, conducted in accordance with the STROBE reporting guidelines, was performed at the Pediatric Allergy and Immunology Clinic of Istanbul Kanuni Sultan Süleyman Training and Research Hospital. A total of 161 pediatric patients with serum total IgE ≥ 2000 IU/mL attending the clinic between January 2019 and November 2024 were screened. Thirty-four patients were excluded: those who had reached 18 years of age at the time of initial presentation, those whose medical records were unavailable, those lost to follow-up or with a follow-up duration of less than one year, or those who had fewer than two serum IgE measurements at intervals of at least six months. After applying these criteria, 127 patients with adequate immunological evaluation and follow-up data were enrolled.

Demographic characteristics, date of presentation, serum total IgE levels (baseline and follow-up measurements), eosinophil percentage and absolute eosinophil count (percentage eosinophilia defined as >4% and absolute eosinophilia as >500 cells/μL) (both values were documented for the entire cohort), allergy tests (specific IgE), immunoglobulin levels (IgG, IgA, IgM), lymphocyte subsets, and genetic test results (DOCK8, STAT3, whole-exome sequencing, and/or IEI panel) were retrospectively extracted from medical records. Atopic sensitization was assessed by specific IgE measurement to a standard inhalant allergen panel comprising Dermatophagoides pteronyssinus (d1), Dermatophagoides farinae (d2), Alternaria alternata, and a grass pollen mixture (GP2 panel: Phleum pratense, Dactylis glomerata, Lolium perenne, Poa pratensis, Festuca pratensis, and Anthoxanthum odoratum). Testing was performed at the discretion of the treating physician based on clinical suspicion of respiratory or cutaneous allergic disease and was therefore not applied systematically across the cohort. Sensitization was defined as a specific IgE ≥ 0.35 kU/L to at least one allergen. The NIH-HIES (National Institutes of Health Hyper-IgE Syndrome) score was calculated using the 21-item clinical scoring system originally described by Grimbacher et al. [5] and subsequently standardized by Woellner et al. [6]. In this system, total scores range from 0 to 100, with ≥40 indicating high likelihood of HIES, 20–39 indeterminate, and <20 unlikely. Scoring was performed retrospectively by chart review; baseline and follow-up scores were derived from clinical findings documented at the respective time points. In this study, the NIH-HIES score was used not as a diagnostic instrument but as an index reflecting clinical and inflammatory disease burden. In patients with a pre-existing allergic diagnosis (asthma, allergic rhinitis, atopic dermatitis, or chronic urticaria), serum total IgE measurement was performed as part of the routine clinical evaluation of the underlying allergic disease. In patients without a pre-existing allergic diagnosis, the specific indication for initial IgE measurement could not always be reconstructed from retrospective chart review; these patients had typically been referred to the pediatric allergy clinic for evaluation of symptoms or findings considered potentially allergic in origin by the referring physician. Concurrent medications at the time of IgE measurement were also reviewed. No patient was receiving systemic immunosuppressive therapy or systemic corticosteroids during the study period; concurrent treatment was limited to topical preparations, oral antihistamines, and inhaled corticosteroids where indicated for the underlying allergic disease.

Intestinal parasitosis was diagnosed by stool microscopy (including direct examination and cellophane tape preparation), macroscopic identification of parasites in stool specimens (reported by a family member or documented by the clinician), and serological testing (enzyme-linked immunosorbent assay [ELISA] and/or indirect hemagglutination assay [IHA]) when indicated. Hydatid cyst was diagnosed based on serological methods (IHA and/or ELISA). Scabies was diagnosed by clinical examination, dermoscopic evaluation, and/or family history (concurrent pruritus and rash in household contacts). Caregiver-reported household contact history and macroscopic parasite identification were documented as supportive diagnostic evidence. Subtype classification of parasitic infections was not routinely performed owing to technical limitations.

Parasitological investigation was performed at the discretion of the treating physician based on symptoms (abdominal complaints, perianal pruritus, unexplained eosinophilia), family contact history, or migration/travel background rather than through systematic cohort-wide screening. In cases where caregivers provided direct visual evidence of parasites or ova in stool (photographs or video documentation reviewed by the clinician) in combination with a positive household contact history, the diagnosis was considered clinically established and confirmatory microscopy was not routinely required—reflecting standard outpatient pediatric practice in endemic settings where definitive visual identification is considered sufficient for treatment initiation. Patient-by-patient coverage of each testing modality is reported in the Results.

Genetic evaluation was performed at the discretion of the treating physician based on clinical judgment and experience, without pre-defined criteria given the retrospective design. STAT3 and DOCK8 gene analyses were ordered as first-line tests where indicated, with a multi-gene IEI panel and/or clinical exome sequencing (CES) performed in selected patients. Statistical analyses were performed using SPSS version 22.0 (IBM, Chicago, IL, USA). Continuous variables are expressed as median and interquartile range (IQR); categorical variables as frequencies and percentages. Between-group comparisons were made using the Mann–Whitney U test, and within-group baseline-to-follow-up comparisons using the Wilcoxon signed-rank test; *p* < 0.05 was considered statistically significant. Effect size was reported as the Hodges–Lehmann (HL) location shift estimate with 95% confidence intervals (CIs) derived from full pairwise bootstrap resampling (5000 iterations), alongside Cohen r. The primary outcome was the between-group difference in IgE reduction percentage; secondary and exploratory outcomes included IgE levels falling below 2000 IU/mL, change in NIH-HIES score, and component analysis. No correction for multiple comparisons was applied to secondary analyses; a Bonferroni threshold of *p* < 0.017 was separately calculated for the three-component analysis. Multivariable linear regression was used to examine predictors of IgE reduction percentage (independent variables: presence of parasitic infection, log-transformed baseline IgE, age, and follow-up duration); logistic regression was used to assess the independent association with IgE declining below 2000 IU/mL. Model fit was evaluated using the Hosmer–Lemeshow test, Nagelkerke R^2^, and the area under the receiver operating characteristic curve (AUC-ROC). Linear regression assumptions were formally verified; homoscedasticity was confirmed by the Breusch–Pagan test and absence of multicollinearity by variance inflation factor (VIF) analysis. A pre-specified sensitivity analysis was conducted using only objectively documented parasitic infections, defined as stool microscopy or cellophane tape positivity, serological confirmation (IHA), or direct macroscopic identification of parasites or ova in stool (clinician-verified photographic/video documentation provided by caregivers), and excluding the clinically diagnosed scabies subgroup (*n* = 22).

The study was approved by the Institutional Ethics Committee of Istanbul Kanuni Sultan Süleyman Training and Research Hospital (approval no: KAEK/2024.11.247; approval date: 27 November 2024). The most recent clinical record included in this study is dated 2 November 2024. All data were retrospectively extracted from existing clinical records solely after IRB approval was granted; no patient data were accessed for research purposes prior to that date.

## 3. Results

A total of 127 patients were enrolled (78 male, 49 female). Median age was 6.3 years (IQR: 3.7–9.9), and 80.3% of patients were between 3 and 12 years of age. Median follow-up duration was 2.4 years (IQR: 1.9–2.9). Median serum total IgE at presentation was 4076 IU/mL (IQR: 3190–5833). Percentage eosinophilia (>4%) was present in 93 patients (73.2%) and absolute eosinophilia (>500 cells/μL) in 77 patients (60.6%). Median baseline eosinophil percentage was 8.3% (IQR: 4.0–12.9) and median absolute eosinophil count was 651 cells/μL (IQR: 336–994). Specific IgE testing was performed in 105 patients (82.7%), of whom 85 (81.0%) were sensitized to at least one allergen. The most frequent allergic diagnoses were allergic rhinitis (45.7%), atopic dermatitis (23.6%), asthma (20.5%), and chronic urticaria (7.9%). The baseline clinical, etiologic, and immunological characteristics of the cohort are summarized in Table 1 and Table 2. Etiologic evaluation identified parasitic infections in 31 unique patients (24.4%), distributed as follows: 18 patients (14.2%) with intestinal parasitosis only, 9 (7.1%) with scabies only, 2 (1.6%) with concurrent intestinal parasitosis and scabies, and 2 (1.6%) with hydatid cyst only. Kawasaki disease was also identified in two patients (1.6%); these were included in the non-parasitic group, and comparative analyses were therefore conducted between the parasitic (*n* = 31) and non-parasitic (*n* = 96) groups.

Diagnostic method distribution is as follows. Among the 20 patients with intestinal parasitosis, 7 were diagnosed by stool microscopy, 3 by cellophane tape preparation, and 10 by macroscopic identification of parasites or ova in stool (documented by caregiver photograph/video or direct clinician visualization), supplemented by household contact history; these 10 patients did not undergo confirmatory microscopy. Both hydatid cyst patients were diagnosed by serology (IHA). Cohort-wide, stool microscopy was performed in 25 patients (19.7%; 7 positive, 18 negative), cellophane tape testing in 3 (2.4%), and parasite serology (IHA) in 2 (1.6%). Within the non-parasitic group (*n* = 96), 18 patients (18.8%) underwent stool microscopy with negative results; the remaining 78 patients (81.3%) did not undergo routine parasitological testing.

The NIH-HIES score across the entire cohort had a median of 16.0 points (IQR: 13.0–19.0). Scores remained below 20 in 108 patients (85.0%) and fell in the indeterminate range (20–39) in 19 patients (15.0%); no patient reached the ≥40-point threshold. Among the 19 patients with baseline NIH-HIES score ≥20, 7 (36.8%) were in the parasitic group and 12 were (63.2%) in the non-parasitic group; none of these 19 patients reached the ≥40-point threshold at baseline or follow-up.

Genetic evaluation was performed in 40 patients (31.5%), including DOCK8 analysis in 16, STAT3 analysis in 12, clinical exome sequencing in 7, and an IEI gene panel in 11; some patients underwent more than one type of test. Patients who underwent genetic testing had significantly higher baseline IgE levels [6065 (4023–10,257) IU/mL], eosinophil percentages, and NIH-HIES scores [19 (16.0–20.2)] compared with those who did not (all *p* < 0.001). Among evaluated patients, DOCK8 deficiency was confirmed in only one patient (2.5%); the remaining patients had negative or inconclusive (variant of uncertain significance, VUS) results. By depth of evaluation, 18 patients (14.2% of the cohort) underwent comprehensive testing (CES and/or a multi-gene IEI panel), 18 patients (14.2%) underwent targeted testing only (STAT3 and/or DOCK8 without broader panel analysis), and 4 patients were referred for genetic evaluation without documented completion of testing. Accordingly, comprehensive IEI exclusion was available for a minority of the cohort (14.2%), and the remaining patients were evaluated against a narrower set of candidate genes or were not genetically evaluated at all.

Clinical and atopic characterization of the non-parasitic group (*n* = 96) revealed a predominantly atopic phenotype. Atopic dermatitis (documented as a non-zero NIH-HIES eczema component) was present in 19 patients (19.8%); 58 patients (60.4%) carried at least one atopic diagnosis (asthma, allergic rhinitis, atopic dermatitis, or chronic urticaria), and 26 patients (27.1%) had two or more atopic diagnoses consistent with an atopic march pattern. Among the 71 non-parasitic patients tested for specific IgE, 62 (87.3%) were sensitized to at least two of the five tested allergens (polysensitization), and 14 (19.7%) were sensitized to four or five allergens, reflecting a heavily atopic phenotype sufficient to explain the degree of IgE elevation observed. Among the 38 non-parasitic patients without a formal atopic diagnosis, 26 had specific IgE testing performed, and 23 of these (88.5%) nevertheless exhibited polysensitization on specific IgE testing, suggesting subclinical atopic sensitization contributing to elevated total IgE. No patient in the cohort carried a diagnosis of cystic fibrosis or documented allergic bronchopulmonary aspergillosis (ABPA). These findings indicate that the non-parasitic group is composed predominantly of atopic or polysensitized patients in whom total IgE elevation is biologically expected, rather than patients with isolated allergic rhinitis or asthma without an atopic substrate.

**Figure 1:**
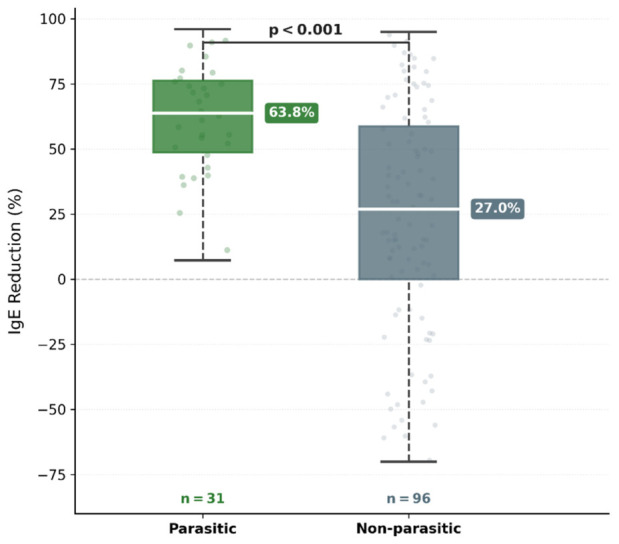
Distribution of IgE reduction percentage in parasitic and non-parasitic groups. Boxes represent median and interquartile range (IQR); whiskers indicate 1.5 × IQR boundaries; dots represent individual values. The dashed horizontal line denotes the zero reference. IgE reduction (%) = (baseline − follow-up IgE)/baseline IgE × 100. Mann–Whitney U test.

At baseline, no statistically significant differences were observed between the parasitic (*n* = 31) and non-parasitic (*n* = 96) groups with respect to IgE levels, eosinophil percentage, or NIH-HIES score (all *p* > 0.05; Table 3). At follow-up, IgE levels were significantly lower in the parasitic group [1793 (1085–2009) IU/mL vs. 3037 (1560–4796) IU/mL; *p* = 0.005]. The primary outcome—median IgE reduction percentage—was 63.8% (IQR: 48.7–76.3) in the parasitic group and 27.0% (IQR: 0.0–58.8) in the non-parasitic group, a difference that was highly significant (*p* < 0.001; Figure 1). During follow-up, IgE levels fell below the enrollment threshold of 2000 IU/mL in 23 parasitic-group patients (74.2%), compared with 35 of 96 (36.5%) in the non-parasitic group (*p* < 0.001). Follow-up eosinophil percentage was significantly lower in the parasitic group (2.9% vs. 4.0%; *p* = 0.044); the same pattern was confirmed using absolute eosinophil count (238 vs. 290 cells/μL; *p* = 0.044). Baseline absolute eosinophil counts were comparable between groups (630 vs. 672 cells/μL; *p* = 0.873), consistent with the baseline percentage comparison. Follow-up NIH-HIES score was also significantly lower in the parasitic group (median 11 vs. 13; *p* = 0.005).

In multivariable linear regression, the presence of parasitic infection was independently associated with IgE reduction percentage after adjusting for baseline IgE level, age, and follow-up duration (B = 27.49; 95% CI: 14.08–40.91; *p* < 0.001; R^2^ = 0.123). In logistic regression, parasitic infection was independently associated with IgE levels declining below 2000 IU/mL at follow-up (odds ratio [OR] = 8.26; 95% CI: 2.76–24.68; *p* < 0.001), with baseline IgE emerging as the dominant predictor in the model (*p* < 0.001). Model fit was satisfactory (Hosmer–Lemeshow χ^2^ = 2.91, *p* = 0.940; Nagelkerke R^2^ = 0.577; AUC = 0.816).

Component-level analysis of the NIH-HIES score revealed that the IgE component declined in 23 (74.2%) and 35 (36.5%) patients in the parasitic and non-parasitic groups, respectively (*p* < 0.001). The eosinophil component declined in 24 (77.4%) versus 39 (40.6%) patients (*p* < 0.001). Eczema component regression was significantly more frequent in the parasitic group (25.8% vs. 4.2%; *p* = 0.001). The median NIH-HIES score decrease was 6.0 points (IQR: 3.0–8.5) in the parasitic group and 2.0 points (IQR: 0.0–6.0) in the non-parasitic group (*p* < 0.001).

A pre-specified sensitivity analysis was performed to address differential diagnostic certainty between objectively documented and clinically diagnosed cases. The parasitic group was restricted to objectively documented cases (*n* = 22; intestinal parasitosis confirmed by stool microscopy or serology, and hydatid cyst confirmed by serology), excluding clinically diagnosed scabies-only cases. In this restricted cohort (*n* = 118), median IgE reduction was 60.0% (IQR: 45.7–75.2) in the lab-confirmed parasitic group versus 27.0% (IQR: 0.0–58.8) in the non-parasitic group (Mann–Whitney *p* = 0.003; Hodges–Lehmann shift 23.4, 95% CI: 7.9–43.3). Lab-confirmed parasitic etiology remained an independent predictor of IgE reduction in multivariable linear regression after adjustment for log-transformed baseline IgE, age, and follow-up duration (B = 22.49; 95% CI: 6.90–38.08; *p* = 0.005), and of IgE normalization below 2000 IU/mL in logistic regression (OR = 6.59; 95% CI: 2.04–21.30; *p* = 0.002; Hosmer–Lemeshow χ^2^ = 1.81, *p* = 0.986; Nagelkerke R^2^ = 0.327; AUC = 0.790). The preservation of effect magnitude and statistical significance across all primary and secondary endpoints indicates that the primary finding is not driven by clinically diagnosed scabies cases.

Among the 31 parasitic-group patients, 8 (25.8%) underwent genetic evaluation; all returned negative or inconclusive (VUS) results, with no confirmed IEI.

In the exploratory subgroup of seven parasitic-group patients with a baseline NIH-HIES score ≥ 20, median IgE declined from 5202 to 1890 IU/mL, and median eosinophil percentage and NIH-HIES score also declined (all Wilcoxon signed-rank *p* = 0.016, reflecting the uniform directionality of change in all seven paired comparisons); six of seven patients (85.7%) crossed below the 20-point indeterminate threshold at follow-up. This observation is derived from a small sample and should be regarded as hypothesis-generating.

## 4. Discussion

Atopic disease dominated the etiologic landscape, with sensitization identified in 81.0% of tested patients—in keeping with the 71.6% and 77% reported by Metbulut et al. and Joshi et al. in comparable pediatric series [1,2]. Polysensitization was particularly frequent (87.3% of tested non-parasitic patients sensitized to ≥2 allergens), and multiple concurrent atopic diagnoses were present in 27.1% of the non-parasitic group—a cumulative atopic burden that is biologically sufficient to drive total IgE above 2000 IU/mL in the absence of parasitic or monogenic etiologies. No patient carried a diagnosis of cystic fibrosis or documented ABPA. The low confirmed IEI rate (0.8%), well below the 5.8% of Metbulut et al., is best explained by the composition of our cohort. Our center did not serve as an IEI referral hub during the study period; patients were identified on the basis of IgE elevation alone rather than prior clinical suspicion of IEI, capturing a broad, unselected population in whom atopic and infectious etiologies predominate. The NIH-HIES score distribution supports this interpretation: no patient reached the ≥40-point threshold and the cohort median of 16 points reflects a case mix with inherently low pre-test probability for primary immunodeficiency. Genetic testing was reserved for the highest-risk subgroup, yet even within that enriched stratum the confirmed IEI rate was only 2.5%, consistent with genuine rarity rather than underascertainment [1]. A recent pediatric cohort similarly reported that non-atopic causes accounted for only 2% of children with elevated IgE [7]. This interpretation nonetheless rests on a subset of the cohort rather than on comprehensive evaluation of all patients, and residual undiagnosed IEI in the untested majority cannot be formally excluded. The low confirmed IEI rate is in itself consistent with the central clinical observation of this study: in an unselected population of children with markedly elevated IgE, the prior probability of monogenic IEI is low and the majority of indeterminate NIH-HIES scores reflect non-IEI etiologies, including parasitic infection.

Given the selective parasitological investigation described above, the observed prevalence should be interpreted as a minimum estimate rather than a precise population-level rate. The parasitic infection rate of 24.4% stands in sharp contrast to the 1.5% reported by Joshi et al. at a North American tertiary center [2]. The most plausible explanation is demographic: Istanbul has experienced sustained rural-to-urban migration, and many parasitically infected families in our cohort came from regions with higher endemic parasite exposure. The Belhassen-García data—showing parasite-associated eosinophilia in 10–50% of immigrant children referred to a tropical medicine unit—suggest that populations shaped by sustained migration carry a substantially higher parasitic burden than those reported from established Western tertiary centers [8].

Both helminthic and ectoparasitic infestations—including scabies—skew the host immune response toward a Th2 phenotype, driving IgE synthesis and peripheral eosinophilia [9,10,11]. In patients with heavy or prolonged infestation, IgE levels can reach striking heights: Roberts et al. reported a median of 17 times the upper limit of normal in crusted scabies [12]. Secondary excoriations and cutaneous inflammation may also fulfil the eczema criterion of the NIH-HIES score. Together, these three features—elevated IgE, eosinophilia, and eczema—constitute the low-specificity cluster that both Woellner et al. (sensitivity 75%, specificity 80.1% for STAT3 mutation) and Schimke et al. have flagged as diagnostically ambiguous [6,13,14]. Öztürk et al.’s case report of NIH-HIES score regression after antiparasitic therapy in a child evaluated for IEI is, in retrospect, a single-patient preview of the pattern we observed across 31 patients [15]. We used the NIH-HIES score throughout as a longitudinal index of inflammatory burden rather than a diagnostic classifier—a distinction that matters for interpreting what followed. The similar baseline scores in both groups (*p* = 0.518) are consistent with the interpretation that any subsequent divergence reflects follow-up trajectory rather than pre-existing differences.

The parasitic group encompasses clinically and pathophysiologically distinct entities: scabies is an ectoparasitic infestation that differs in clinical presentation, anatomical site, and treatment approach from intestinal helminthiases and hydatid disease [16], although all three can drive elevated IgE through Th2-biased immune activation. Intestinal helminths drive sustained Th2 polarization through mucosal antigen sampling; scabies elicits cutaneous IgE responses that can reach very high titers, particularly in crusted forms [10,12]; and hydatid cyst produces a more variable humoral response depending on cyst viability. Despite these differences, Th2 skewing with IgE amplification is a mechanistic feature shared across categories [9,10], which supports grouped analysis for the reversible-phenocopy hypothesis while acknowledging the inherent heterogeneity. The pre-specified sensitivity analysis excluding clinically diagnosed scabies supports, but does not fully prove, the robustness of the primary finding.

At follow-up, parasitic-group patients showed a substantially greater IgE reduction (Table 3) that held after multivariable adjustment and remained significant in the sensitivity analysis excluding scabies (*p* = 0.003). The most straightforward confound would be differential atopic treatment: if the non-parasitic group had received more intensive therapy, one would expect the IgE gap to narrow over time, not widen. The comparable atopic disease rates and sensitization proportions between groups (Table 3) argue against this. The roughly eight-fold higher odds of IgE normalization below 2000 IU/mL (OR = 8.26) in the parasitic group, combined with the absence of any patient reaching the ≥40-point threshold, is more consistent with a population in whom parasite-driven inflammation was the principal IgE amplifier than with a HIES cohort showing atypical recovery.

The component-level data add texture to this picture. All three inflammatory NIH-HIES components—IgE, eosinophil percentage, and eczema—regressed more often in the parasitic group (all *p* ≤ 0.01; Table 3), whereas infection-related components (pneumonia, skin abscesses, candidiasis) were unchanged in both groups. This pattern is mechanistically coherent: the structural and anatomical features of the scoring system (high-arched palate, characteristic facies, scoliosis, retained dentition, joint hyperextensibility) cannot change regardless of treatment, so any score movement must originate from the inflammatory items. The more pronounced eczema regression in the parasitic group most likely reflects resolution of scabies-related cutaneous inflammation. None of this, of course, amounts to proof of causation: we cannot rule out contributions from concurrent atopic therapy or natural disease course, and the low R^2^ (0.123) of the multivariable model underlines that parasitic etiology is one driver among several.

## 5. Limitations

Several limitations of this study deserve acknowledgment. Parasitological testing was not performed systematically (documented in 30 of 127 patients, 23.6%), introducing detection bias: the reported 24.4% prevalence represents a minimum estimate within a clinically evaluated subset rather than a population-level rate. The parasitic category pooled helminthic, ectoparasitic, and cystic infections that differ in immune activation patterns. Scabies diagnosis rested on clinical and dermoscopic criteria, carrying a misclassification risk; intestinal helminth subtyping was not routinely performed. Th17 cell measurements were not available, limiting our ability to apply the full Woellner diagnostic framework. Although no patient received systemic immunosuppressive therapy, inhaled corticosteroids and antihistamines may exert modest effects on eosinophil trafficking or total IgE dynamics, and the absence of dose-level individual medication data precludes formal adjustment. Similarly, specific IgE testing was performed in 105 of 127 patients (82.7%) at the discretion of the treating physician rather than systematically, which may have biased upward the reported atopic sensitization rate (81.0% of tested patients) if clinicians preferentially ordered testing in children with overt allergic features. The timing of post-treatment IgE measurements was not standardized; values were obtained at variable intervals according to the treating clinician’s judgment.

Genetic evaluation was performed in only 31.5% of the cohort, and comprehensive testing (clinical exome sequencing and/or a multi-gene IEI panel) was completed in only 14.2%. The remaining patients were either tested with a targeted approach limited to STAT3 and DOCK8 or were not genetically evaluated at all. Negative or inconclusive results in a subset of the cohort cannot be extrapolated to all patients, and the non-parasitic group should not be interpreted as definitively free of underlying inborn errors of immunity—it is more appropriately described as a clinically low-suspicion group in whom IEI was not identified within the scope of the testing performed. Negative genetic results do not definitively exclude IEI, and the gene panels used may not capture all known IEI-associated variants; VUS results in particular cannot be conclusively interpreted. Patients with persistent clinical features therefore require long-term follow-up regardless of negative results.

## 6. Conclusions

In children presenting with IgE ≥ 2000 IU/mL, parasitic infections account for roughly one in four cases and produce a clinical phenotype that overlaps closely with HIES. This overlap can mislead clinical scoring systems; in our cohort, 8 of 31 parasitic-group patients underwent genetic evaluation and none had a confirmed IEI. In populations with substantial parasitic prevalence, considering parasitological evaluation in children with elevated IgE and indeterminate clinical scores may represent a useful adjunct to the diagnostic process, particularly when the clinical phenotype is otherwise ambiguous. This approach is not proposed as a replacement for genetic evaluation where clinically indicated, and its generalizability to populations with lower parasitic burden remains to be determined in prospective studies.

## Figures and Tables

**Table 1:** Clinical and etiologic characteristics of the study cohort (*n* = 127).

Variable	n (%) or Median (IQR)
Male	78 (60.9%)
Age, years	6.3 (3.7–9.9)
Follow-up duration, years	2.4 (1.9–2.9)
Baseline IgE (IU/mL)	4076 (3190–5833)
Follow-up IgE (IU/mL)	2316 (1434–4202)
Baseline eosinophil (%)	8.3 (4.0–12.9)
Eosinophilia >4%	93 (73.2%)
**Allergic diseases**	
Atopic sensitization (of 105 patients tested)	85 (81.0%)
Allergic rhinitis/Asthma/Atopic dermatitis/Chronic urticaria	58 (45.7%)/26 (20.5%)/30 (23.6%)/10 (7.9%)
**Parasitic etiology**	
Intestinal parasitosis only	18 (14.2%)
Scabies only	9 (7.1%)
Intestinal parasitosis + scabies	2 (1.6%)
Hydatid cyst only	2 (1.6%)
Kawasaki disease	2 (1.6%)
Total parasitic (unique patients)	31 (24.4%)
Genetic evaluation performed/Confirmed IEI	40 (31.5%)/1 (0.8%)
**NIH-HIES score**	
Median (IQR)	16.0 (13.0–19.0)
<20 points/20–39 points	108 (85.0%)/19 (15.0%)

Values are expressed as median (IQR) or n (%). Kawasaki disease was not classified within the parasitic/infectious group and is presented separately. IEI: inborn errors of immunity; NIH-HIES: National Institutes of Health Hyper-IgE Syndrome.

**Table 2:** Immunological parameters of the study cohort (*n* = 127).

Parameter	Median (IQR)
**Lymphocyte subsets (%)**	
CD3	71.3 (67.7–74.4)
CD4	39.1 (34.5–44.8)
CD8	24.3 (22.1–27.3)
CD19	14.7 (11.9–19.6)
**Immunoglobulins (mg/dL)**	
IgG	1109 (976–1308)
IgA	135 (89–186)
IgM	117 (92–158)

CD subsets and immunoglobulin levels were assessed in all patients as part of routine immunological evaluation. Values are expressed as median (IQR).

**Table 3:** Comparison of parasitic and non-parasitic groups.

Variable	Parasitic (*n* = 31)	Non-Parasitic (*n* = 96)	*p*	HL Shift (95% CI)	r
Baseline IgE (IU/mL)	3856 (3215–5623)	4079 (3193–5902)	0.860	−68 (−690; 797)	0.02
**Follow-up IgE (IU/mL)**	1793 (1085–2009)	3037 (1560–4796)	0.005 **	−1123 (−1660; −411)	0.25
**IgE reduction (%)**	63.8 (48.7–76.3)	27.0 (0.0–58.8)	<0.001 ***	29.3 (12.7; 46.5)	0.35
Baseline eosinophil (%)	7.9 (4.6–12.1)	8.4 (3.9–13.0)	0.926	−0.1 (−1.9; 1.7)	0.01
Follow-up eosinophil (%)	2.9 (0.8–4.0)	4.0 (1.6–8.0)	0.044 *	−1.4 (−2.6; 0.0)	0.18
NIH-HIES score, baseline	16 (14–19)	16 (13–19)	0.518	0.0 (−1.0; 2.0)	0.06
**NIH-HIES score, follow-up**	11 (8–14)	13 (10–16)	0.005 **	−3.0 (−4.0; −1.0)	0.25
**IgE < 2000 IU/mL at follow-up**	23 (74.2%)	35 (36.5%)	<0.001 ***	—	—
Allergic rhinitis/Asthma/Atopic dermatitis/Chronic urticaria	17 (54.8%)/7 (22.6%)/11 (35.5%)/1 (3.2%)	41 (42.7%)/19 (19.8%)/19 (19.8%)/9 (9.4%)	0.301/0.799/0.090/0.449	—	—
Atopic sensitization (of tested patients)	20/24 (83.3%)	65/81 (80.2%)	1.000	—	—
Genetic evaluation performed	8 (25.8%)	32 (33.3%)	—	—	—
Confirmed IEI	0 (0%)	1 (1.0%)	—	—	—

* *p* < 0.05, ** *p* < 0.01, *** *p* < 0.001; Mann–Whitney U, chi-square, or Fisher exact test. HL: Hodges–Lehmann location shift; 95% CI: derived from full pairwise bootstrap resampling (5000 iterations); r: Cohen effect size (r < 0.3 small, 0.3–0.5 moderate, >0.5 large). IgE reduction (%): (baseline − follow-up IgE)/baseline IgE × 100. The Bonferroni threshold for the three-component analysis is *p* < 0.017; all component *p*-values meet this criterion. Values are expressed as median (IQR) or n (%).

## Data Availability

The datasets generated and analyzed during the current study are not publicly available due to patient privacy regulations but are available from the corresponding author on reasonable request.
